# Continuous and non-invasive thermography of mouse skin accurately describes core body temperature patterns, but not absolute core temperature

**DOI:** 10.1038/s41598-020-77786-5

**Published:** 2020-11-26

**Authors:** Vincent van der Vinne, Carina A. Pothecary, Sian L. Wilcox, Laura E. McKillop, Lindsay A. Benson, Jenya Kolpakova, Shu K. E. Tam, Lukas B. Krone, Angus S. Fisk, Tatiana S. Wilson, Tomoko Yamagata, James Cantley, Vladyslav V. Vyazovskiy, Stuart N. Peirson

**Affiliations:** 1 Department of Physiology and Genetics, Sleep and Circadian Neurosciences Institute, University of Oxford, Oxford, UK; 2 Nuffield Department of Clinical Neurosciences, Sleep and Circadian Neurosciences Institute, University of Oxford, Oxford, UK; 3 Department of Neurobiology, Brudnick Neuropsychiatric Research Institute, University of Massachusetts Medical School, Worcester, MA USA; 4 Division of Systems Medicine, School of Medicine, University of Dundee, Dundee, UK; 5 Department of Biology, Williams College, Williamstown, MA USA

**Keywords:** Physiology, Animal physiology

## Abstract

Body temperature is an important physiological parameter in many studies of laboratory mice. Continuous assessment of body temperature has traditionally required surgical implantation of a telemeter, but this invasive procedure adversely impacts animal welfare. Near-infrared thermography provides a non-invasive alternative by continuously measuring the highest temperature on the outside of the body (T_skin_), but the reliability of these recordings as a proxy for continuous core body temperature (T_core_) measurements has not been assessed. Here, T_core_ (30 s resolution) and T_skin_ (1 s resolution) were continuously measured for three days in mice exposed to ad libitum and restricted feeding conditions. We subsequently developed an algorithm that optimised the reliability of a T_skin_-derived estimate of T_core_. This identified the average of the maximum T_skin_ per minute over a 30-min interval as the optimal way to estimate T_core_. Subsequent validation analyses did however demonstrate that this T_skin_-derived proxy did not provide a reliable estimate of the absolute T_core_ due to the high between-animal variability in the relationship between T_skin_ and T_core_. Conversely, validation showed that T_skin_-derived estimates of T_core_ reliably describe temporal patterns in physiologically-relevant T_core_ changes and provide an excellent measure to perform within-animal comparisons of relative changes in T_core_.

## Introduction

Body temperature is a key physiological parameter that affects a host of physiological processes and can be utilised as a scientific and humane endpoint in biomedical research^1–4^. Despite its physiological relevance, body temperature is often ignored in rodent studies because of the practical difficulties associated with its measurement; especially when continuous measurements are required.

The measurement of body temperature in small rodents is typically performed by contact method, implanted telemeter or thermal imaging^5^. Each of these methods is associated with pros and cons^5^. Contact measurements such as inserting a rectal temperature probe are relatively easy to perform and do not require expensive equipment but are associated with an increased body temperature due to handling stress for the animal, potential health complications due to probe insertion, and only provide a snapshot of a continuously changing variable. Telemeter implantation enables the continuous and accurate recording of core body temperature (T_core_) but requires invasive surgery resulting in stress, may alter physiology, requires substantial time and skill from the researcher, and malfunctions of the telemeter can typically not be remedied. Thermal imaging and other non-contact temperature measurements provide a non-invasive method to record body temperature^6^, but these methods typically require animal handling, are not continuous, and do not measure T_core_^7–18^. The non-invasive nature of thermography measurements does however provide the potential to assess body temperature in undisturbed freely-moving laboratory mice, removing the confounding factor of handling stress and representing an obvious refinement in terms of animal welfare.

Here, we develop and optimise an algorithm for processing thermal imaging data of freely-moving mice with the goal of assessing whether the resulting T_core_ estimate, based on continuous measurements of the highest temperature on the outside of the body (T_skin_), can be used to describe (changes in) T_core_. Mice were implanted intraperitoneally with a body-temperature telemeter while T_skin_ was recorded every second by thermal imaging for three days. This was done under standard laboratory conditions as well as in a subgroup of food-restricted mice exhibiting daily torpor, a transient hypometabolic state associated with a marked decrease in body temperature^3^. Assessment of different algorithm parameters (summary statistics, sampling and averaging intervals) identified averaging of the maximum T_skin_ per 60 s over 30-min intervals (T_skin,max_) as the most reliable way to estimate T_core_. T_skin,max_ provides an accurate description of relative changes in T_core_ within individual animals. Between-animal variation in the relationship between T_core_ and T_skin,max_ does however limit the utility of T_skin_ measurements as a measure of absolute within-animal changes in T_core_ or differences in T_core_ between animals.

## Results

### Estimating core body temperature non-invasively by continuously recording skin temperature

**Figure 1:**
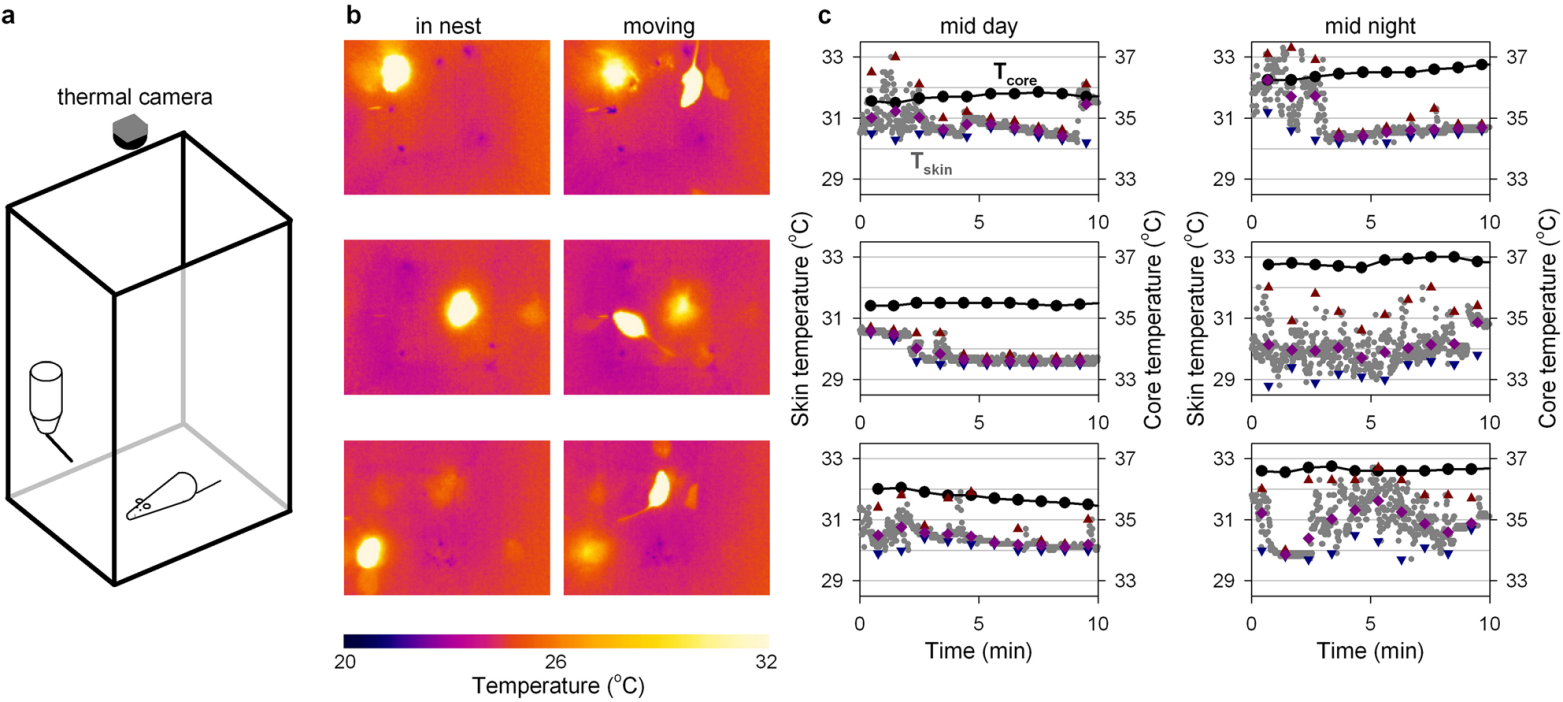
Continuous recording of skin (T_skin_) and core temperature (T_core_) in freely-moving laboratory mice. (**a**) Mice were each individually housed in an open-topped cage placed under a thermal camera. (**b**) Thermal images of three mice at rest (left) and while moving through the cage (right). Skin temperature was recorded by storing the temperature of the warmest pixel in view (1 Hz). (**c**) Representative 10-min traces of T_core_ (1 min^−1^, black dots) and T_skin_ (1 Hz, grey dots) for three mice in the middle of the light- (left) and dark-phase (right). The distribution of T_skin_ measurements within each minute is quantified by the minimal (blue), mean (pink) and maximal (red) T_skin_. Large fluctuations in T_skin_ can be observed especially at night, likely as a result of variability in the warmest observed pixel due to movement of the animal.

Measuring T_skin_ using near-infrared thermography enables the continuous assessment of body temperature during both day and night in freely-moving animals. Here, T_skin_ was recorded in five wildtype mice housed in open-topped cages at an ambient temperature of 22 ± 1 °C (Fig. 1a). A limited amount of nesting material was provided, to ensure that the mice were fully visible at all times. As expected, the warmest spot in each image was associated with the location of the mouse in the cage (Fig. 1b), thus enabling the description of T_skin_ by recording the temperature of the warmest pixel each second (Fig. 1c). As illustrated in the representative 10-min recordings (Fig. 1c), T_skin_ often changed rapidly (< 1 min) by 1–2 °C while the simultaneously recorded T_core_ did not reveal corresponding changes. Based on observations of the mice during these recordings, we established that these rapid changes in T_skin_ were typically associated with movement of the animal. The observed T_skin_ was typically higher and more variable during movement, likely as a result of changes in the exposed parts of the skin due to the animal’s change in position and posture (Fig. S1). Consistent with this interpretation, periods of high T_skin_ variability were more common during the night when mice are most active. The high variability of T_skin_ compared to T_core_ (Fig. 1c) highlights the importance of processing T_skin_ measurements to obtain a reliable proxy for T_core_ rather than relying on raw T_skin_ measurements.

### Reducing skin temperature variability by optimising algorithm parameters

**Figure 2:**
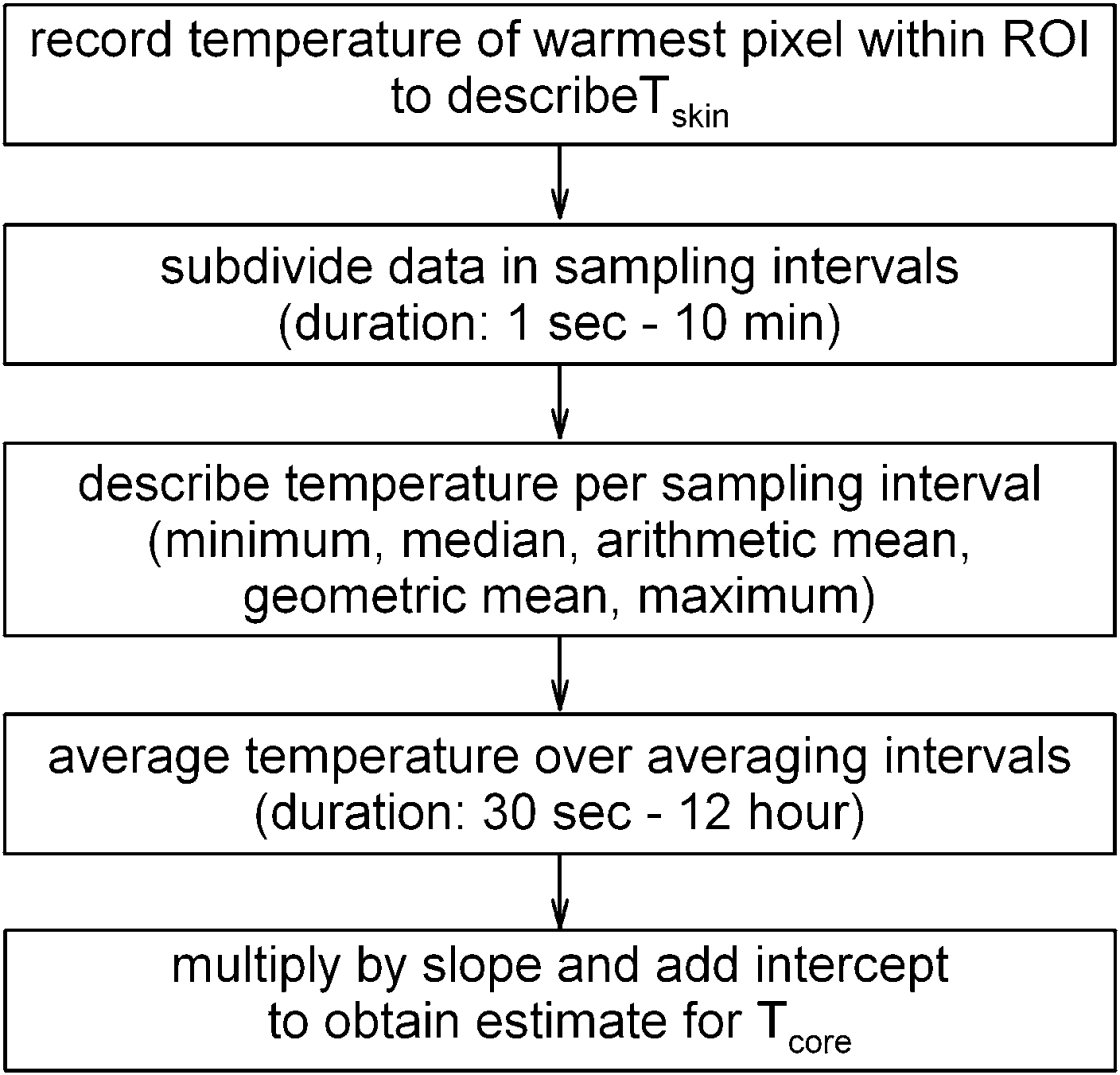
Dataflow used to optimise the estimation of T_core_ based on T_skin_ measurements.

The algorithm developed here was designed to estimate T_core_ based on T_skin_ measurements taken every second. For this, a summary statistic was used to describe T_skin_ during each short sampling interval (1 s–10 min) and these values were averaged over a longer averaging interval (30 s–12 h). This averaged measure of T_skin_ (T_skin,max_) was subsequently transformed to obtain an estimate of T_core_ using the slope and intercept describing the linear relationship between T_skin,max_ and T_core_ (Fig. 2). The present paper describes the optimisation of algorithm parameters with the objective of estimating T_core_ with high accuracy, equal variance at different levels of T_core_, and ideally a relationship between T_skin,max_ and T_core_ with a slope of 1 (i.e. T_core_ = T_skin,max_ + constant).

**Figure 3:**
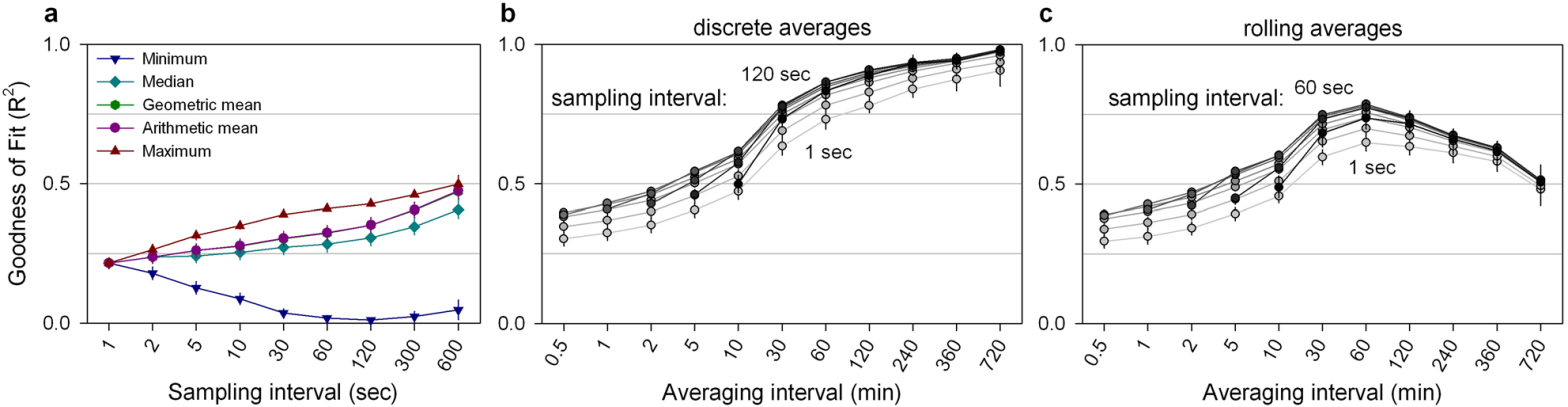
Optimisation of algorithm parameters used to estimate T_core_ based on thermal camera measurements of T_skin_. (**a**) Goodness of fit associated with different summary statistics calculated over intervals between 1 s and 10 min. The outcomes associated with the arithmetic mean and geometric mean are indistinguishable. (**b**) Goodness of fit associated with discrete estimates of T_core_ for each averaging-interval duration based on T_skin,max_ over different sampling intervals (1–600 s) and averaged over intervals between 30 s and 12 h. Fill and line colour become progressively darker with increasing sampling interval duration. Sampling of T_skin,max_ over intermediate sampling intervals (30–120 s) consistently resulted in a better description of T_core_ compared to shorter and longer sampling intervals. Sampling T_skin,max_ over an intermediate sampling interval resulted in an ~ 15% improvement of fit compared to taking the mean temperature (i.e. 1 s sampling interval) over the same averaging interval. (**c**) Goodness of fit associated with estimating each measurement of T_core_ (30 s time resolution) using a rolling average based on T_skin,max_ over different sampling intervals (1–600 s) and averaged over intervals between 30 s and 12 h. Fill and line colour become progressively darker (lightest: 1 s → darkest: 600 s) with increasing sampling interval duration. Data represents the between-individual mean and SEM goodness of fit associated with the presented combination of algorithm parameters.

The optimal algorithm parameters were determined by assessing how the possible parameter combinations (sampling interval, summary statistic, averaging interval) affected the reliability of the T_core_ estimate. The optimal sampling interval and summary statistic for estimating T_core_ based on T_skin_ measurements was determined by comparing the goodness of fit associated with each combination of algorithm parameters (Fig. 3a). Using the minimal T_skin_ per sampling interval to estimate T_core_ resulted in a progressively worse fit with increasing sampling interval length while all other summary statistics resulted in an improved fit with longer sampling intervals. In all five mice, use of the maximum temperature as a summary statistic resulted in a better fit of T_core_ compared to the median, arithmetic- or geometric mean, especially at intermediate sampling interval lengths (Fig. 3a, Fig. S2). The superiority of using the maximum per sampling interval as opposed to calculating the arithmetic mean over the whole averaging interval is illustrated in subsequent analyses (Fig. 3b,c) by the improved goodness of fit associated with different sampling intervals compared to the 1-s interval (since the T_skin_ sampling rate was also 1 s, the 1-s sampling interval estimate is equivalent to taking the mean over all measurements within an averaging interval). The optimal sampling interval also depended on the chosen averaging interval with sampling intervals of 60 s or 120 s resulting in the best estimate of T_core_ while both shorter and longer sampling intervals were associated with a reduced goodness of fit (Fig. 3b,c). The quality of the T_core_ estimate was strongly influenced by the length of the averaging interval (Fig. 3b,c). The accuracy of discrete T_core_ estimates increased consistently with longer averaging intervals in all five individual mice although the most pronounced increase occurred between averaging intervals of 10 and 60 min (Fig. 3b). The increasing accuracy by which progressively longer (> 60 min) discrete averaging intervals estimated the mean T_core_ over that same (long) interval (Fig. 3b) was however inherently coupled with a decreasing ability to describe T_core_ changes over time (Fig. S3). The optimisation of this trade-off between the accuracy of the average and describing T_core_ changes over time was done by sliding the averaging interval in 30 s steps to estimate a rolling average for T_core_ (Fig. 3c). This analysis demonstrated that averaging intervals of 30 or 60 min maximised the accuracy of the average and the description of the temporal changes in T_core_. Furthermore, an averaging interval of 30 min resulted in a relationship between T_skin,max_ and T_core_ with an average slope close to 1 (Fig. S4). Based on these outcomes, we conclude that the optimal algorithm to estimate T_core_ based on T_skin_ measurements taken every second samples T_skin,max_ per 60 s and averages these values over 30 min intervals.

### Relationship between skin and core temperature

**Figure 4:**
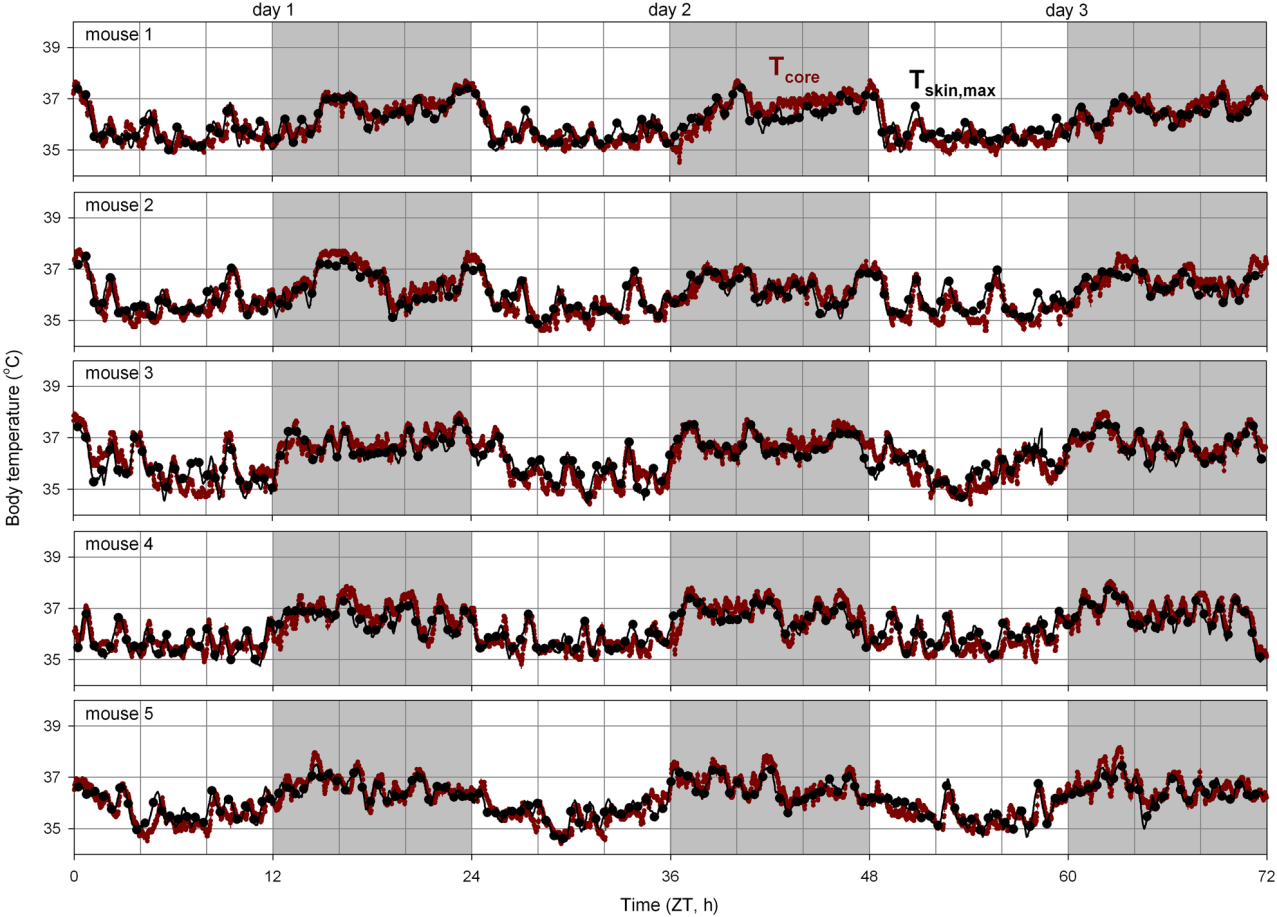
Three-day core temperature recordings measured directly (T_core_, red) and estimated based on skin temperature (T_skin,max_, black) in five mice. Core temperature estimates based on T_skin,max_ are depicted as a rolling average (black line) and as discrete averages (black dots, 1 per 30 min). T_skin,max_ was calculated by averaging the maximum T_skin_ per minute over a 30-min interval. Slope and intercept describing the linear relationship between T_skin,max_ and T_core_ were optimised for each mouse individually. Day and night are represented by the white- and light-grey background, respectively. As expected for a nocturnal species, both T_core_ and T_skin,max_ measurements show that body temperature is highest during the night in mice. ZT: Zeitgeber time.

Combining the T_skin_-derived estimate obtained using the algorithm described above with the slope and intercept describing the linear relationship between T_skin,max_ and T_core_ optimised for each individual mouse resulted in an excellent description of T_core_ over the three-day test period (Fig. 4). Such an individualised optimisation does however require the implantation of a telemeter, thus negating the main benefit of using non-invasive thermal imaging to estimate T_core_. Our goal here is to describe the average relationship between T_skin,max_ and T_core_ and assess whether these group-level parameters enable an adequate estimation of T_core_ based on T_skin_ measurements in individual mice.

**Figure 5:**
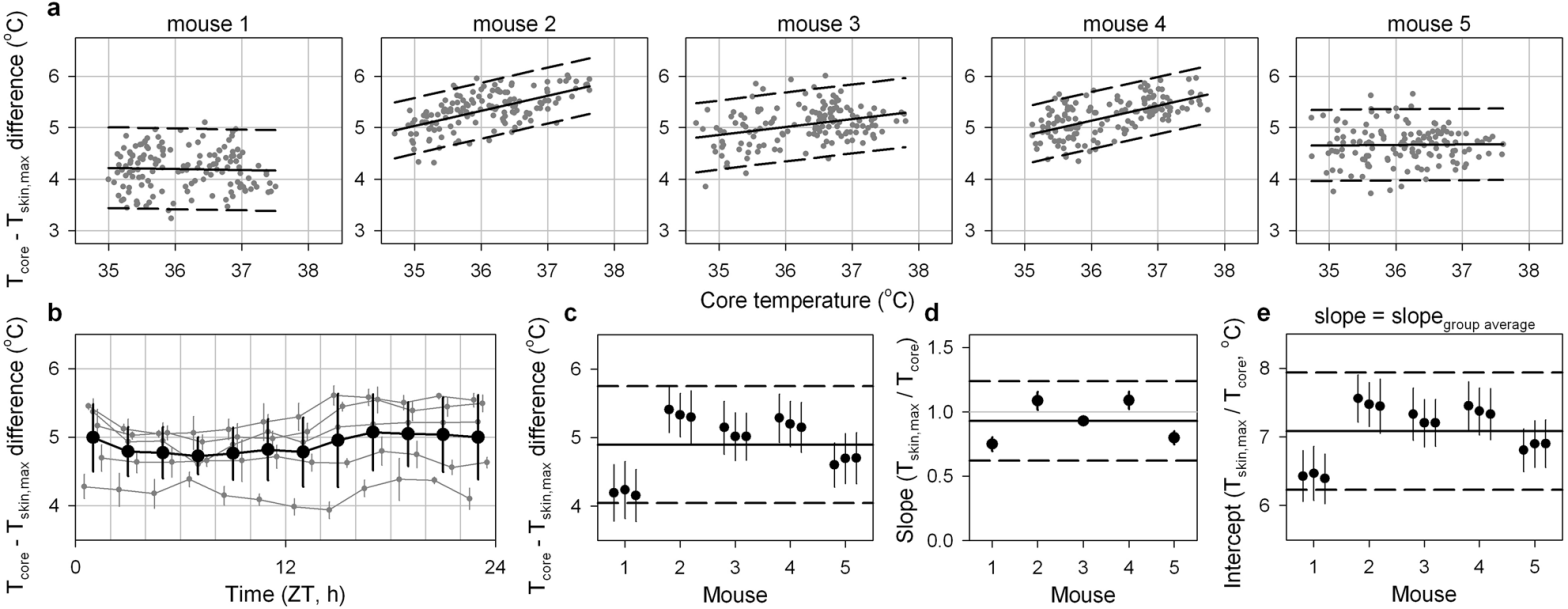
Core temperature (T_core_) estimation by continuous skin temperature (T_skin,max_) measurements; accuracy assessment. (**a**) Correlations of T_core_ with the difference between T_core_ and T_skin,max_ for all five mice. Comparisons are made between the average T_core_ per 30 min and the average of the maximum T_skin_ per minute over the same averaging interval. Solid lines represent the least-squares linear fit while dashed lines enclose the 2-standard-deviations area surrounding this fit. (**b**) The difference between T_core_ and T_skin,max_ was only marginally correlated with time of day (~ 0.3 °C, *p* < 0.0001). Traces of individual mice (dark grey lines) are slightly offset on the x-axis to improve visibility. (**c**) The difference between T_core_ and T_skin,max_ on each of the three measurement days in all five mice. (**d**) The slope of the relationship between T_core_ and T_skin,max_ in all five mice. Mean and SD summarise the within-individual variance in slope between the three measurement days. (**e**) The intercept of the relationship between T_core_ and T_skin,max_ in all five mice. This assessment incorporated the group-average (0.93) as the slope for all mice. Within-individual variance in the difference between T_core_ and T_skin,max_ (**c**), slope (**d**) and intercept (**e**) was substantially lower than the between-individual variance. Solid lines in (**c**–**e**) represent the group mean while dashed lines enclose the 2-standard-deviations area surrounding this average. Error bars represent SD.

The variance in the difference between T_core_ and T_skin,max_ was independent of T_core_ for all five mice (Fig. 5a), this temperature difference was only minimally influenced by the time of day (range of hourly averages: 4.7–5.1 °C, *p* < 0.0001; Fig. 5b), and the temperature difference was consistent across measurement days within each of the five mice (Fig. 5c). The difference between T_core_ and T_skin,max_ did not correlate with T_core_ in two of the five mice but in the other three mice a significant positive correlation was observed between T_core_ and the difference between T_core_ and T_skin,max_ (Fig. 5a). The relationship between T_core_ and the difference between T_core_ and T_skin,max_ was strongly dependent on the chosen sampling interval but not the averaging interval duration (Fig. S5A). Between-animal variance in this relationship was substantial, however, and precluded the selection of algorithm parameters that would prevent a correlation between T_core_ and the difference between T_core_ and T_skin,max_ in all mice (Fig. S5B). As noted above, the selected algorithm parameters resulted in a relationship between T_core_ and T_skin,max_ with a slope of ~ 1 (Fig. 5d). When this average slope was used to estimate T_core_ based on T_skin,max_, the observed residual difference between T_core_ and T_skin,max_ (intercept) was consistent between days within all mice, although the between-animal variance was substantial (Fig. 5e). Overall, the selected algorithm parameters (averaging maximum T_skin_ per 60 s over 30 min) resulted in an estimate of T_core_ that was highly consistent between days with equal variance at different T_core_ values, a minimised correlation between T_core_ and the difference between T_core_ and T_skin,max_, and a relationship between T_skin,max_ and T_core_ with a slope and intercept of 0.93 and 7.1 °C respectively.

### Between-animal variability in the relationship between skin and core temperature

**Figure 6:**
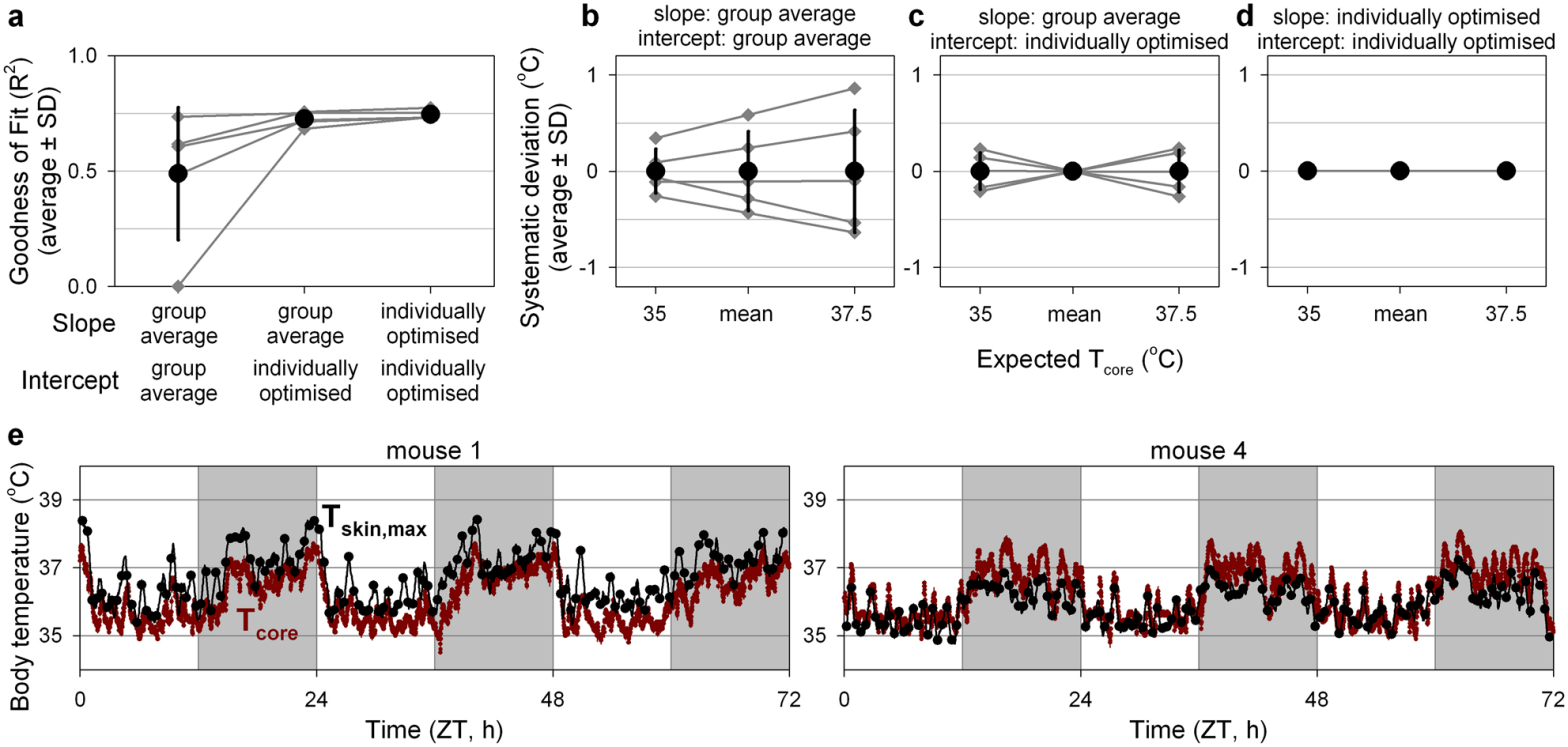
Between-animal variability in the relationship between T_skin,max_ and T_core_ limits utility of T_skin_-derived estimates of T_core_. (**a**) Goodness of fit associated with estimating T_core_ based on T_skin,max_ using group average or individually optimised values for the slope and/or intercept in individual mice (dark grey). Group averages are plotted in black. (**b**–**d**) Expected systematic temperature deviations at low, mean and high T_core_ for models using group averages or individually optimised values for the slope and/or intercept in individual mice (dark grey). Expected systematic deviations are calculated based on the difference between the group-average and individually-optimised slope and intercept for each individual mouse. Group averages are by definition 0, with greater SD values representing higher between-animal variation in the T_core_ estimation error. (**e**) Two representative examples of the measured T_core_ (red) and the estimated temperature based on T_skin,max_ (black) with group averages used as slope and intercept. Core temperature estimates based on T_skin_ are depicted as a rolling average (black line) and as discrete averages (black dots, 1 per 30 min). Day and night are represented by the white- and light-grey background, respectively. Error bars represent SD. ZT: Zeitgeber time.

A key aim of this study was to determine whether thermal imaging could be used to reliably estimate T_core_ non-invasively in freely-moving mice. To this end, it would be essential that T_core_ can be estimated without having to determine the relationship between T_core_ and T_skin,max_ for each individual animal. As a minimal assessment of this requirement, the group-average slope and intercept were used to estimate T_core_ based on T_skin,max_ in the five mice for which these group averages had been optimised. The use of the group average slope and intercept dramatically reduced the quality of T_core_ estimates in some of the mice (Fig. 6a) because it resulted in a systematic under- or overestimation of T_core_ (Fig. 6e) due to between-animal differences in slope and intercept. Changing of algorithm parameters could not further reduce the between-animal variance in slope and intercept (Figs. S4B, S6). As a result of the high between-animal variance in the relationship between T_core_ and T_skin,max_ observed in the current group of five mice, T_skin,max_ did not provide a reliable estimate of the absolute value of T_core_ in individual mice (systematic deviation range: − 0.6 to + 0.9 °C; Fig. 6b). To place these values in context, these deviations span approximately half the observed T_core_ range (3.1–3.8 °C; Fig. 4). Conversely, between-animal comparisons of absolute changes in T_core_ based on T_skin,max_ could be made with greater accuracy (systematic deviation range: − 0.5 to + 0.5 °C per 2.5 °C T_core_ change; Fig. 6c). Within-animal comparisons of relative changes in T_core_ could be estimated with the highest accuracy (systematic deviation: 0 °C, within-animal day-to-day intercept range: 0.2 °C, within-day intercept SD: 0.3–0.4 °C; Figs. 5e, 6d), thus demonstrating the utility of thermography for comparisons of relative T_skin_ changes between days (or treatments) within animals.

### Estimating core body temperature during daily torpor

**Figure 7:**
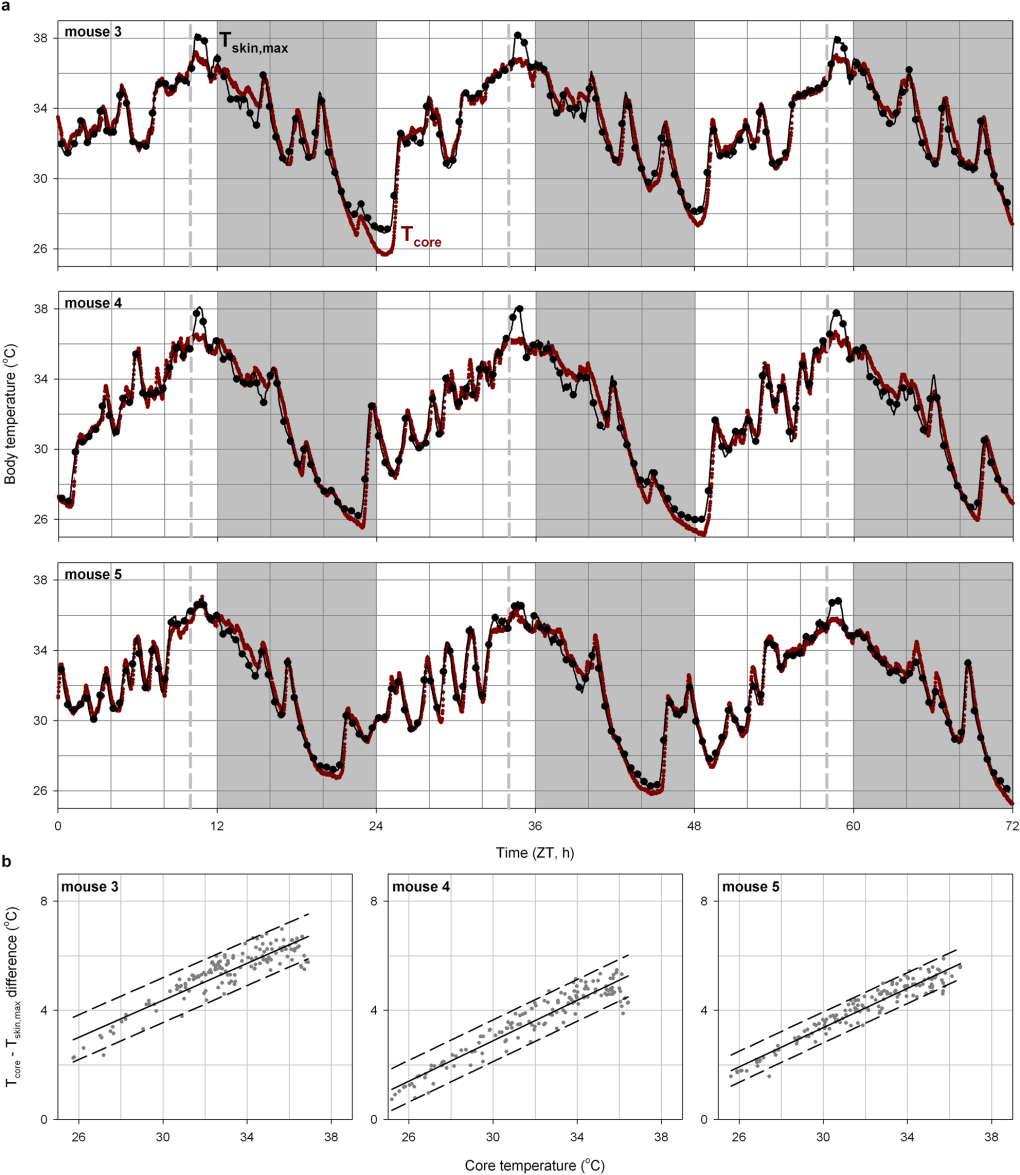
Body temperature during daily torpor in mice exposed to food restriction. (**a**) Three-day core temperature recordings measured directly (T_core_, red) and estimated based on skin temperature (T_skin,max_, black) of three mice during food restriction. Daily torpor was induced by chronic food restriction to ~ 70% of ad libitum food intake. Food was provided daily, three hours before lights-off (dashed line). Day and night are represented by the white- and light-grey background, respectively. Skin temperature was calculated by averaging the maximum T_skin_ per minute over a 30-min interval. Slope and intercept describing the linear relationship between T_skin,max_ and T_core_ was optimised for each mouse individually. ZT: Zeitgeber time. (**b**) The difference between T_core_ and T_skin,max_ was strongly correlated with T_core_ in all three individual mice. Comparisons are made between the average T_core_ per 30 min and T_skin,max_ over the same averaging interval. Solid lines represent the least-squares linear fit while dashed lines enclose the 2-standard-deviations area surrounding this fit.

Exposure to energetically challenging conditions (e.g. hunger, cold) induces energy saving strategies such as daily torpor in mice^3^. Here, food intake of three mice was restricted to a single daily meal consisting of ~ 70% of their ad libitum intake resulting in daily torpor bouts in all mice (duration: 4–8 h, minimum core temperature: 25–27 °C; Fig. 7a). In line with our findings in mice fed ad libitum, averaging T_skin,max_ per 60 s over a 30-min interval resulted in an accurate estimate of T_core_ (Fig. S7A–C) with comparable variance at different values of T_core_ (Fig. 7b). The relationship between T_core_ and T_skin,max_ had a slope that was consistently higher than that observed in homeothermic mice (Fig. S7D,E), reflecting an altered relationship between T_core_ and T_skin,max_ in mice under energetically challenging conditions. The difference between T_core_ and T_skin,max_ decreased linearly with lower values of T_core_ (Fig. 7b), thus complicating the T_skin_-derived estimation of T_core_ (i.e. slope > 1). This correlation between T_core_ and the difference between T_core_ and T_skin,max_ could not be eliminated by altering algorithm parameters (Fig. S7F,G). Although the sample size was insufficient to reliably estimate between-animal variance in the relationship between T_core_ and T_skin,max_, the observed difference in slopes in individual mice (range: 1.42–1.53, Fig. S8) would translate to systematic deviations of ± 0.5 °C between mice over the 10 °C temperature difference observed under these energetically challenging conditions. In line with our observations in ad libitum fed mice, the day-to-day within-animal variance in the relationship between T_core_ and T_skin,max_ was very limited (Fig. S7H–K). This demonstrated the utility of non-invasive continuous thermography measurements to perform within-animal comparisons of relative changes in T_core_ in mice during daily torpor.

## Discussion

Monitoring body temperature provides important information about the physiological and metabolic state of animals. Established techniques for measuring body temperature are associated with restraint stress, do not allow continuous recordings, and/or require complicated invasive surgery^5^. The use of infrared thermography has the potential to provide a non-invasive method to measure T_core_ but its own methodological limitations have to be taken into account^5,6^. Previous applications of non-contact T_skin_ measurements in biomedical research have been limited by the requirement that animals needed to be handled by the experimenter^7–15^, measurements were taken at a limited number of timepoints^7,8,10–17^, and/or measurements resulted in large datafiles requiring complex data analysis^10,12,13,16–18^. The present study developed, optimised and validated an algorithm that enables estimation of relative changes in T_core_ based on the continuous and non-invasive automated measurement of T_skin_ of mice housed at room temperature. The high variability in T_skin_ compared to T_core_ measurements (Fig. 1c) necessitates data processing to obtain a less-variable estimate of T_skin_. Here we show that averaging the maximum T_skin_ per 60 s over a 30 min interval (T_skin,max_) provides the most accurate estimate of T_core_. High between-animal variability in the linear relationship between T_skin,max_ and T_core_ (i.e. slope and intercept) severely limits the accuracy of T_skin_ recordings as a measure of absolute T_core_. Instead, because of the low day-to-day within-animal variability in the relationship between T_skin,max_ and T_core_, T_skin_ recordings provide an excellent tool to assess relative differences in T_core_ within individual animals.

Given the aforementioned strengths and limitations in estimating relative changes in T_core_ based on T_skin_, this method provides an excellent tool to continuously monitor relative T_core_ changes in undisturbed, individually-housed, freely-moving mice. This was illustrated here by characterising the temporal fluctuations in body temperature throughout day and night as well as during daily torpor. The automated and continuous nature of the measurement and data processing steps presented here compare favourably with previous approaches using thermography to assess T_core_ changes^10,12,13,16,17^, albeit at the expense of accuracy of its absolute T_core_ estimate^13,17^. Although the inability to accurately estimate absolute T_core_ values compares negatively to telemeter implantation, this cost will often be outweighed by welfare, time and financial benefits associated with not having to perform surgery, especially in cases where (physiologically-relevant) changes in body temperature are the prime concern^14,16,18^. When used as a humane endpoint, body temperature is often compared to a reference value at a single timepoint^8,11,14^. Although such a between-animal comparison does not suit the current method, the continuous nature of its T_core_ estimate enables welfare decisions to be based on multiple characteristics such as the daily body temperature profile, its timing, and an individually calibrated set point. The requirement that animals are individually housed in open-top cages with reduced access to nesting materials (to ensure visibility of the animal) also provides a limitation of the current approach, although depending on the experimental paradigm this might be a worthwhile trade-off. Overall, we view the method presented here as a useful addition to a repertoire of different approaches to monitor body temperature^5^, that, depending on the specific research question, might provide benefits compared to other established techniques.

## Methods

All animal procedures were approved by the ACER AWERB of the University of Oxford and performed under a UK Home office license in accordance with all relevant laws and regulations. Five wildtype C57Bl6/J mice were implanted intraperitoneally with an Anipill temperature telemeter. Following post-operative recovery mice were housed at an ambient temperature of 22 ± 1 °C in open-top cages, each positioned under a thermal camera. T_skin_ was measured every second by storing the temperature of the warmest pixel. T_core_ was measured every 30 s by the implanted Anipill. The quality of the T_skin_-derived T_core_ estimate was optimised based on the goodness of fit and variance distribution associated with each combination of different summarising statistics (minimum, median, arithmetic mean, geometric mean and maximum), sampling intervals (1 s–10 min), and averaging intervals (30 s–12 h). The linear relationship (slope and intercept) between T_skin,max_ and T_core_ was assessed in 5 ad libitum fed mice and subsequently under energetically challenging conditions in 3 of these mice. Systematic deviations represent the difference between the estimated T_core_ calculated based on individually-optimised versus group-average based descriptions of the relevant relationship between T_skin,max_ and T_core_ for each of the animals and presented assessments. Extended methodological details are available in the *SI Methods* and software templates to calculate T_core_ estimates based on the methods described here have been uploaded to Figshare (10.6084/m9.figshare.12587909).

## Supplementary information

Supplementary information.

## Data Availability

All raw data, scripts and outcomes per individual animal have been uploaded to Figshare (10.6084/m9.figshare.12587495). Software templates (MS Excel, SciLab, Mathlab, R and Python) to estimate core body temperature based on skin temperature measurements can be downloaded from Figshare (10.6084/m9.figshare.12587909).
