# Supplementary material for: Continuous and non-invasive thermography of mouse skin accurately describes core body temperature patterns, but not absolute core temperature
Source: Sci Rep. 2020 Nov 26;10:20680. doi: 10.1038/s41598-020-77786-5 (PMC7693264; doi:10.1038/s41598-020-77786-5)
Supplement: Supplementary file 1 — Supplementary information. [file 41598_2020_77786_MOESM1_ESM.pdf]

Supplemental information

Continuous and non-invasive thermography of mouse skin accurately describes core body temperature patterns, but not absolute core temperature

Vincent van der Vinne, Carina A. Pothecary, Sian L. Wilcox, Laura E. McKillop, Lindsay A. Benson, Jenya Kolpakova, Shu K. E. Tam, Lukas B. Krone, Angus S. Fisk, Tatiana S. Wilson, Tomoko Yamagata, James Cantley, Vladyslav V. Vyazovskiy, Stuart N. Peirson

SI Methods

Animals

All animal procedures were performed under UK Home Office Project License #P828B64BC in accordance with Animal (Scientific Procedures) Act 1986 and the guideline of the University of Oxford. All experimental procedures were reviewed and approved by the central Animal Care and Ethical Review (ACER) Animal Welfare and Ethical Review Body (AWERB) of the University of Oxford.

Five male adult wildtype C57Bl6/J mice (3-6 months old) were obtained from our in-house breeding colony. Mice were group housed (IVC-type cages on sawdust bedding) in same-sex littermate groups from weaning until the start of experimental procedures (12h:12h light-dark cycle, 22 ± 1 °C). Mice had *ad libitum* access to water throughout the experiment. Food (2016 Teklad global 16% protein diet, Envigo, Blackthorne, UK) was available *ad libitum* unless indicated otherwise.

Surgery

Mice were housed individually in a standard IVC cage with Alpha dri bedding (LBS biotech 1032003, Horley, UK) at least 24 h before surgery. Animals were implanted with a temperature telemeter/logger (Anipill v2, Bodycap, Hérouville Saint-Claire, France) in the peritoneal cavity to measure core body temperature. Isoflurane anaesthesia (TEVA UK Ltd, Eastbourne, UK) was induced at 4% and maintained at 1.5-2% in 1.5 L/min 100% O_2. Analgesia and fluid support were provided by subcutaneous injection of meloxicam (0.025 ml per 10 g body weight at 2 mg/ml, Metacam, Boehringer Ingelheim Vetmedica GmbH, Ingelheim/Rhein, Germany), buprenorphine (0.033 ml per 10 g body weight at 0.03 mg/ml, Vetergesic, Ceva Animal Health Ltd, Amersham, UK) and 0.1 ml Saline immediately following anaesthesia induction. Artificial tears were applied to protect the eyes while body temperature (36 °C) was maintained by placement of the animal on a homeostatic temperature regulating system (Harvard Apparatus 50-7221F, Holliston MA, USA) throughout the surgery. The abdominal fur was shaved, the surgical area sterilised by circular wiping with three chloraprep applicators (Enturia ChloraPrep Sterile Sepp Applicator, 0.67 mL, Leawood KS, USA), and the animal covered by a sterile drape with a 2x2 cm opening allowing access to the surgical area. A 1.5 cm caudal-rostral skin incision was made ~5 mm lateral from the abdominal midline, followed by blunt dissection of the skin and muscle layer. Subsequently, a 1 cm caudal-rostral muscle incision was made through the linea alba. The Anipill telemeter was inserted intraperitoneally and the muscle layer closed with a simple continuous suture pattern using a 5-0 absorbable Vicryl suture (Ethicon, Somerville NJ, USA). A splosh of 0.25% bupivacaine hydrochloride anhydrous (Marcaine, Advanz pharma, London, UK) was applied to the outside of the muscle suture to reduce potential internal skin irritation from the suture. The skin was closed by 4-6 simple interrupted inverted sutures (5-0 Vicryl suture) and an additional 0.2 ml saline was given subcutaneously. Post-surgery, mice were allowed to recover in a heated box (28 °C) and

returned to their home cage once locomotor behaviour was observed (after 15-30 min). Post-surgery, analgesia was provided orally (meloxicam in jelly) for at least 3 days while animals were checked 1-2x daily to confirm proper recovery.

Experimental setup and procedures

Following full post-operative recovery, mice were transferred to open-topped recording cages (sawdust bedding) that were each positioned under a thermal camera (Optris PI 160 with standard 61° lens, Optris GmbH, Berlin, Germany). Accurate calibration of the thermal camera was confirmed pre-experimentally by comparison to a common heat source. The camera was positioned above the middle of the cage to ensure that the whole mouse was always in view. Similarly, only a limited amount of nesting material was provided to ensure that the mouse was unable to shield from the overhead camera. Food was provided on the cage floor and replenished regularly under *ad libitum* feeding conditions. T_{skin} was recorded every second for a 3-day period by storing the temperature of the warmest pixel in view using the software provided by the camera's manufacturer (Optris PIX Connect, Optris GmbH). During this experimental period, T_{core} was recorded and stored every 30 s using the standard Anipill recording module. Post-experimental confirmation of the accuracy of the Anipill temperature telemeters/loggers at different temperatures showed that individual device calibration was not required.

The relationship between T_{skin} and T_{core} during daily torpor was assessed in a subgroup of 3 of the 5 mice following the 3-day *ad libitum* feeding condition described above. Daily torpor was induced by restricting daily food intake to a single meal (~70% of *ad libitum*) provided 3 h before lights off (zeitgeber time 9). The exact meal size was calibrated daily based on the body mass (measured daily at lights off) to maintain body mass at 85-90% of *ad libitum* feeding weight. After 1-2 weeks of this torpor-induction protocol, T_{skin} and T_{core} were recorded for multiple days and a 3-day experimental interval during which the mice exhibited daily torpor bouts on each day was selected for each mouse individually.

Data analysis

All data analyses were performed using custom-written scripts in Scilab 6.0.1 (www.scilab.org). Recorded T_{skin} measurements (1 s recording interval) were subdivided in sampling intervals of different durations (range: 1 s - 10 min) with the goal of producing a description of T_{skin} that would produce a better estimate of T_{core} than simply taking the average over the averaging interval. Sampling intervals of 5 s and longer were expressed as a fraction/multiple of the T_{core} sampling interval to compensate for minor differences in sampling interval duration of the T_{core} measurements (range: 28 - 31 s). Sampling/averaging intervals were centred around the timing of T_{core} measurements. T_{core} was assumed to be equal to the nearest measurement for sampling intervals shorter than the T_{core} interval duration while T_{core} was averaged over the full assessment interval for sampling/averaging intervals for longer assessment intervals. The T_{skin} distribution during each sampling interval was described by 5 different summary statistics (minimum, median, arithmetic mean, geometric mean and maximum) which were subsequently assessed to determine the optimal summary statistic to estimate T_{core}. The duration of averaging intervals (30 s - 12 h) was defined as the multiple of the expected number of T_{core} measurements during the chosen interval (duration / 30 s). Discrete averages were calculated by averaging the summary statistics describing all sampling intervals occurring during each of the non-overlapping averaging intervals. Rolling averages were calculated by shifting the averaging window by 30 s for each interval. The quality of T_{skin}-derived T_{core} estimates was assessed based on the associated goodness of fit,

distribution of residuals as well as within- and between-animal variability. As part of these analyses, the optimal slope and intercept describing the linear relationship between $T_{skin,max}$ and T_{core} were estimated for each assessment separately. Group averages of these individually optimised slope and intercept values were subsequently assessed for their ability to estimate T_{core} based on each mouse's $T_{skin,max}$ measurements. The systematic deviation in estimated T_{core} was calculated for each mouse at different body temperatures (low: 35 °C, mean: 36.166 °C, high: 37.5 °C) as the within-individual average deviation resulting from the difference between the individually-optimised and group-average relationships in the part of the relationship between $T_{skin,max}$ and T_{core} that is relevant for the presented assessments (i.e. between-individual absolute differences, within-individual absolute differences, within-individual relative differences). Statistical tests were performed as mixed-effects general liner models with animal# included as a random variable if appropriate while residuals were inspected visually to confirm the assumptions of normality and heterogeneity of variance.

SI Figures

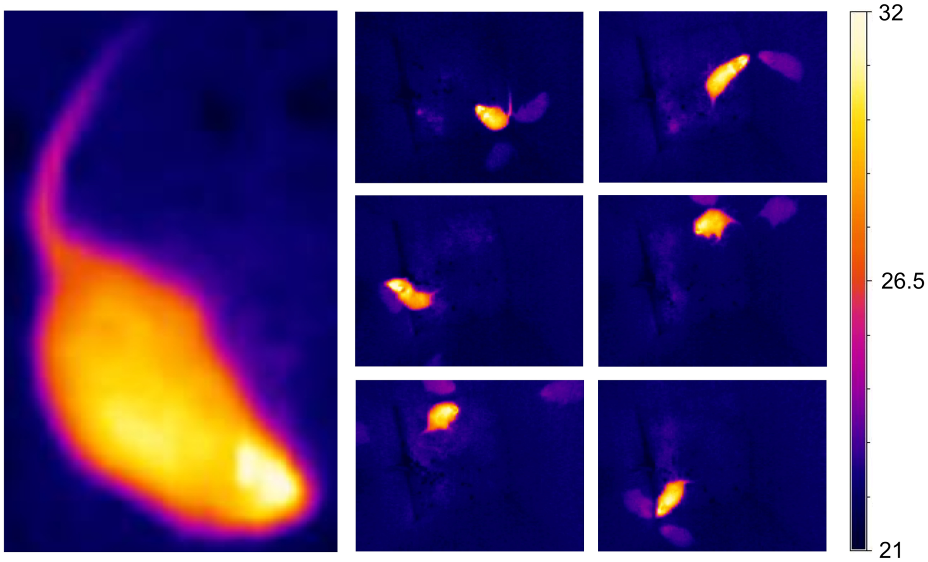

Figure S1: Representative thermal images of a single individually-housed mouse. The warmest spot on the body of the mouse depends on the positioning of the animal relative to the camera but is typically associated with the head or upper back. Reflections of the mouse coming off the walls of the cage can be observed in most images. The colour scale represents temperature in °C.

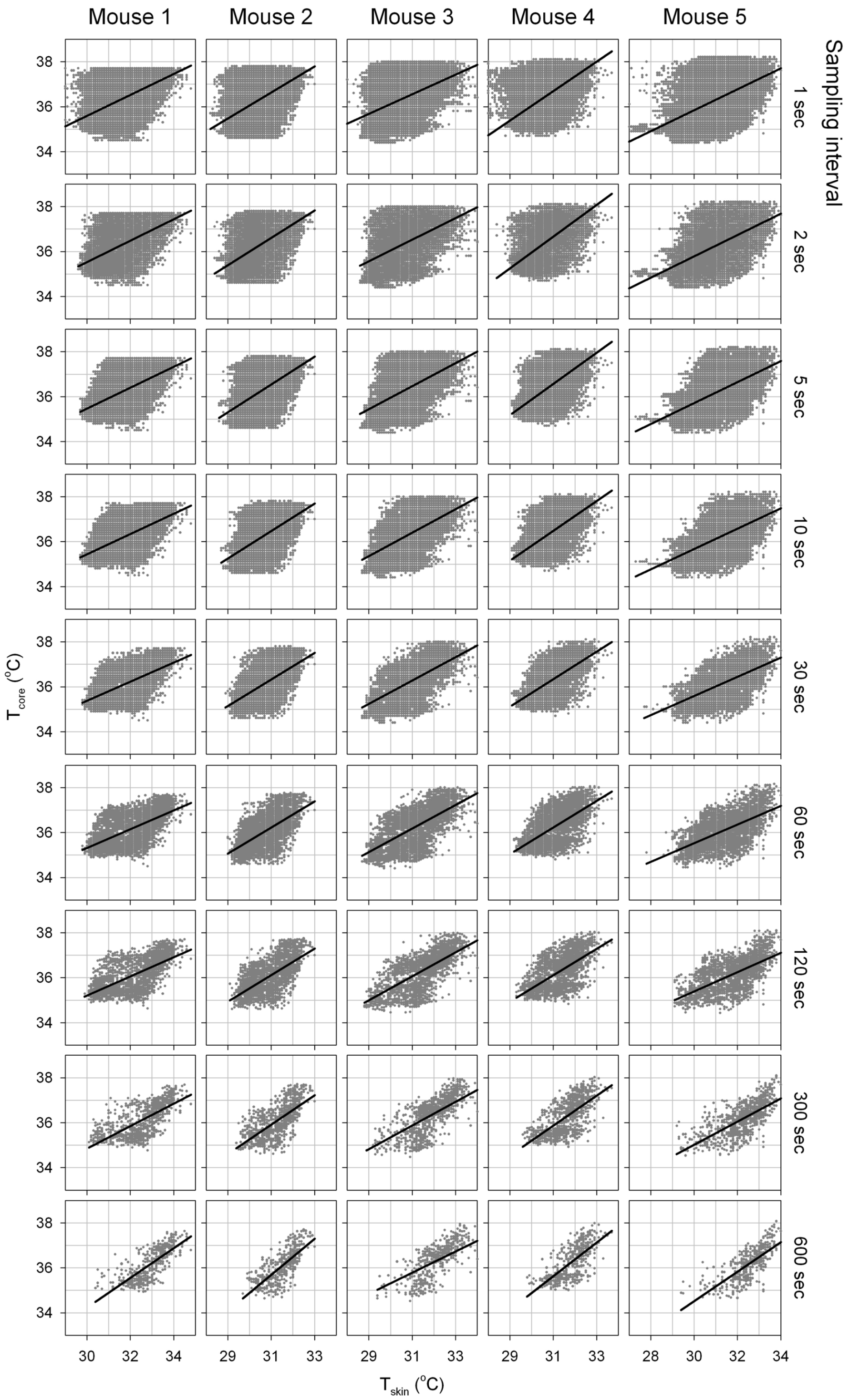

Figure S2: Correlation between T_{skin} and T_{core} for all nine sampling intervals in all five mice. Dark-grey dots represent the correlation between the maximum T_{skin} sampled during each interval and the average T_{core} over that same interval. Black lines represent the least-squares linear fit.

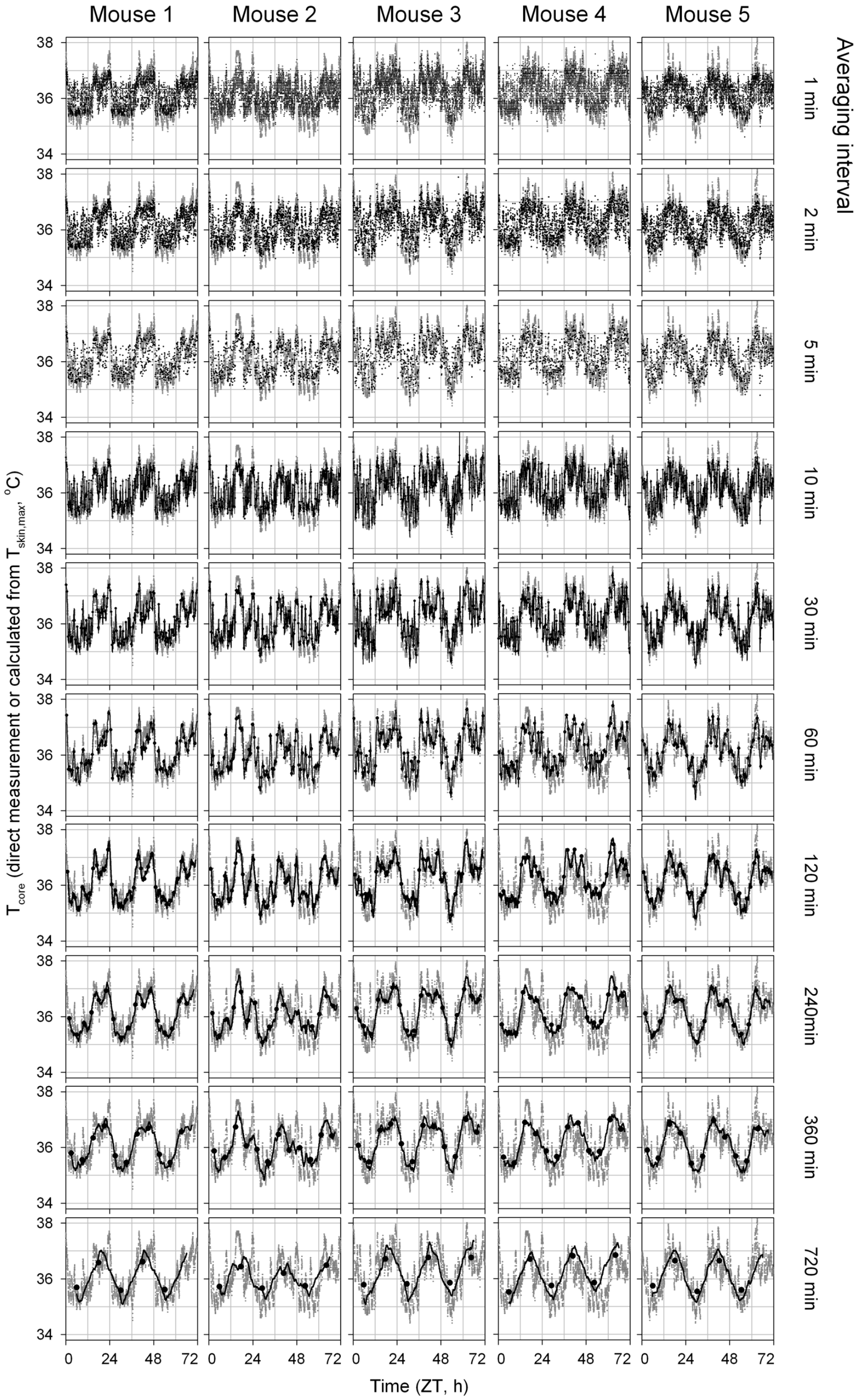

Figure S3: T_{core} measurements and estimates during three-day period for all 10 possible averaging intervals in all five mice. T_{core} was either measured directly (dark-grey dots) or estimated based on $T_{skin,max}$ (discrete averages: black dots, rolling averages: black lines). The maximum T_{skin} was sampled every 60 s and averaged over the specified averaging interval. The relationship between $T_{skin,max}$ and T_{core} (slope and intercept) was optimised for each mouse individually and separately for discrete and rolling averages. ZT: zeitgeber time.

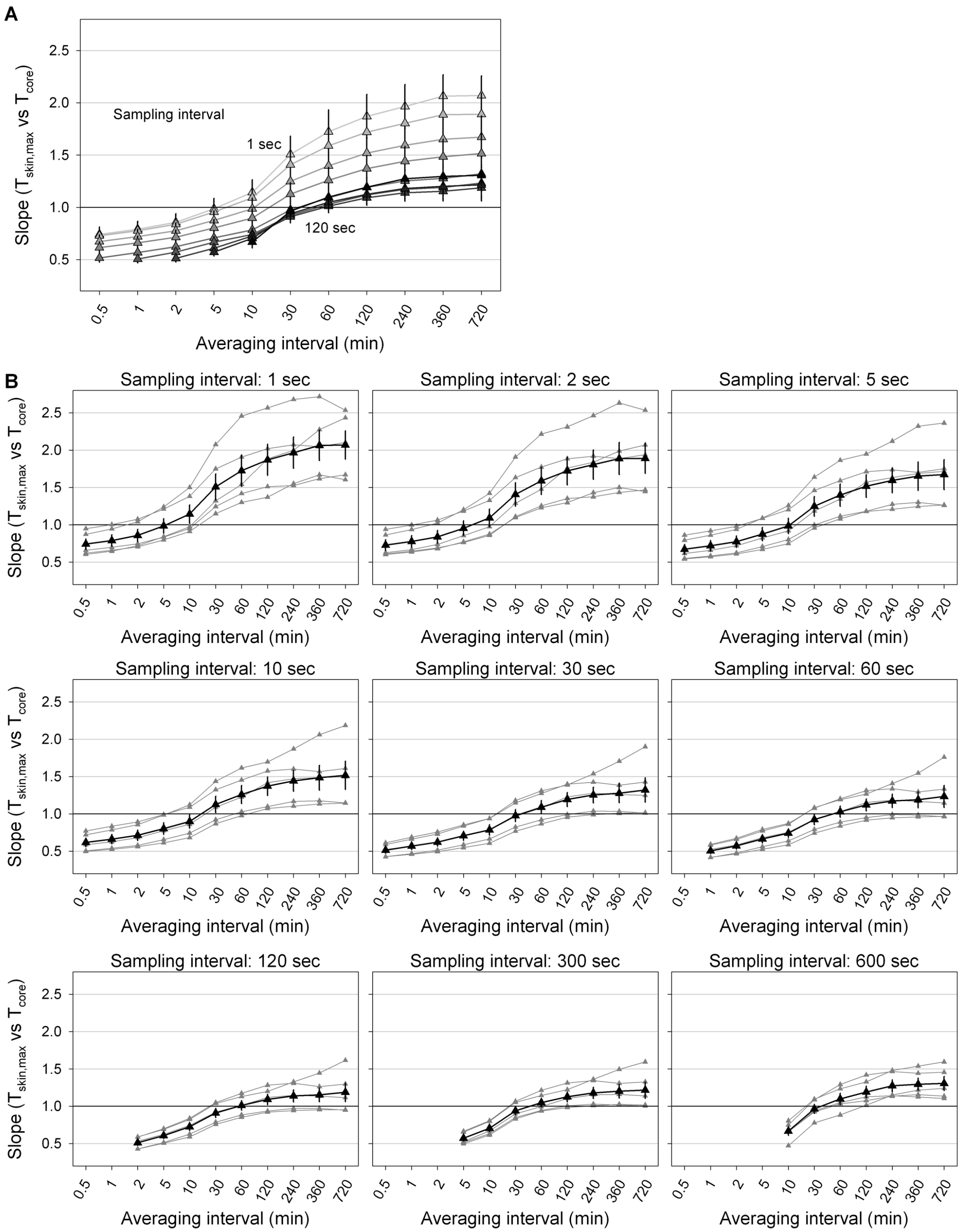

Figure S4: The slope describing the relationship between $T_{skin,max}$ and T_{core}. (A) This slope depends on both the sampling and averaging interval. The slope was minimal for intermediate sampling intervals (60 - 120 s). (B) Between-animal variance in slope for each sampling interval. Dark-grey lines represent the observed slope in each of the five mice. Black lines represent group averages and are identical to data represented in A. $T_{skin,max}$ was used as the summary statistic and all slopes were based on discrete averages.

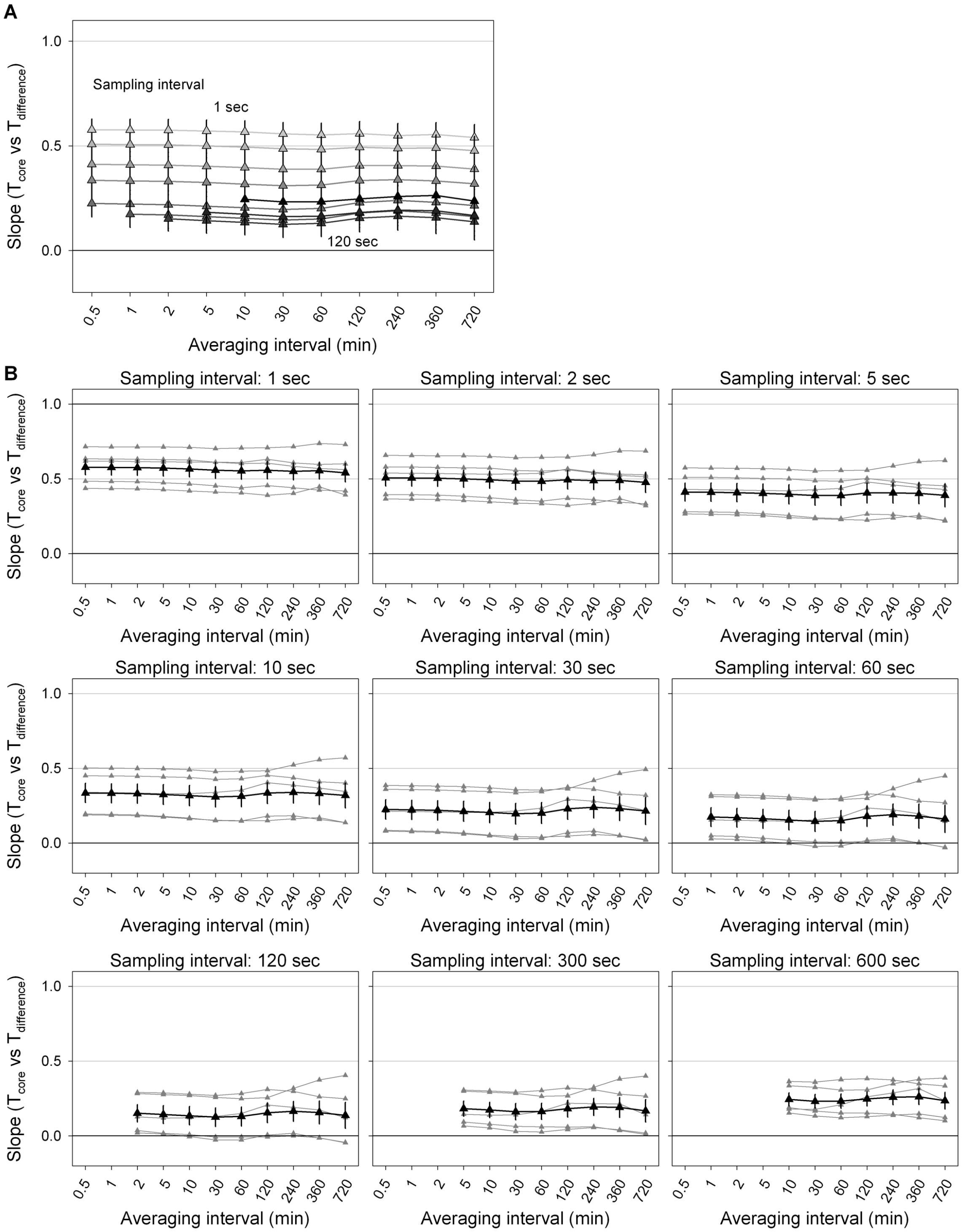

Figure S5: The slope describing the relationship between T_{core} and the difference between $T_{skin,max}$ and T_{core}. (A) This slope depends on the sampling interval but not on the averaging interval. The slope was minimal for intermediate sampling intervals (60 - 120 s). (B) Between-animal differences in slope. Dark-grey lines represent the observed slope in each of the five mice. Black lines represent group averages and are identical to data represented in A. $T_{skin,max}$ was used as the summary statistic and all slopes were based on discrete averages.

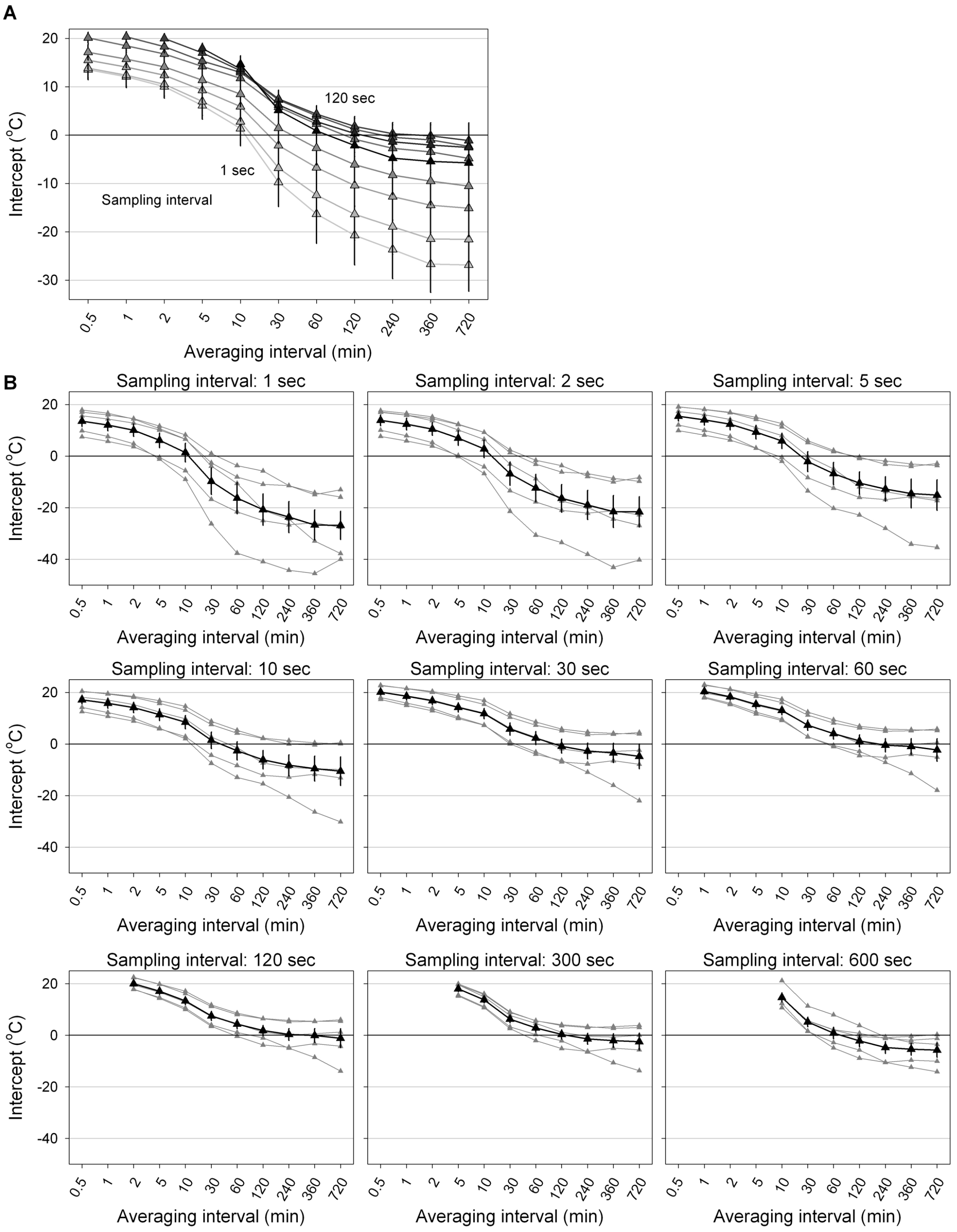

Figure S6: The intercept describing the relationship between $T_{skin,max}$ and T_{core}. (A) This intercept depends on both the sampling and averaging interval. The intercept was maximal for intermediate sampling intervals (60 - 120 s). (B) Between-animal variance in intercept for each sampling interval. Dark-grey lines represent the observed intercept in each of the five mice. Black lines represent group averages and are identical to data represented in A. $T_{skin,max}$ was used as the summary statistic and all slopes were based on discrete averages.

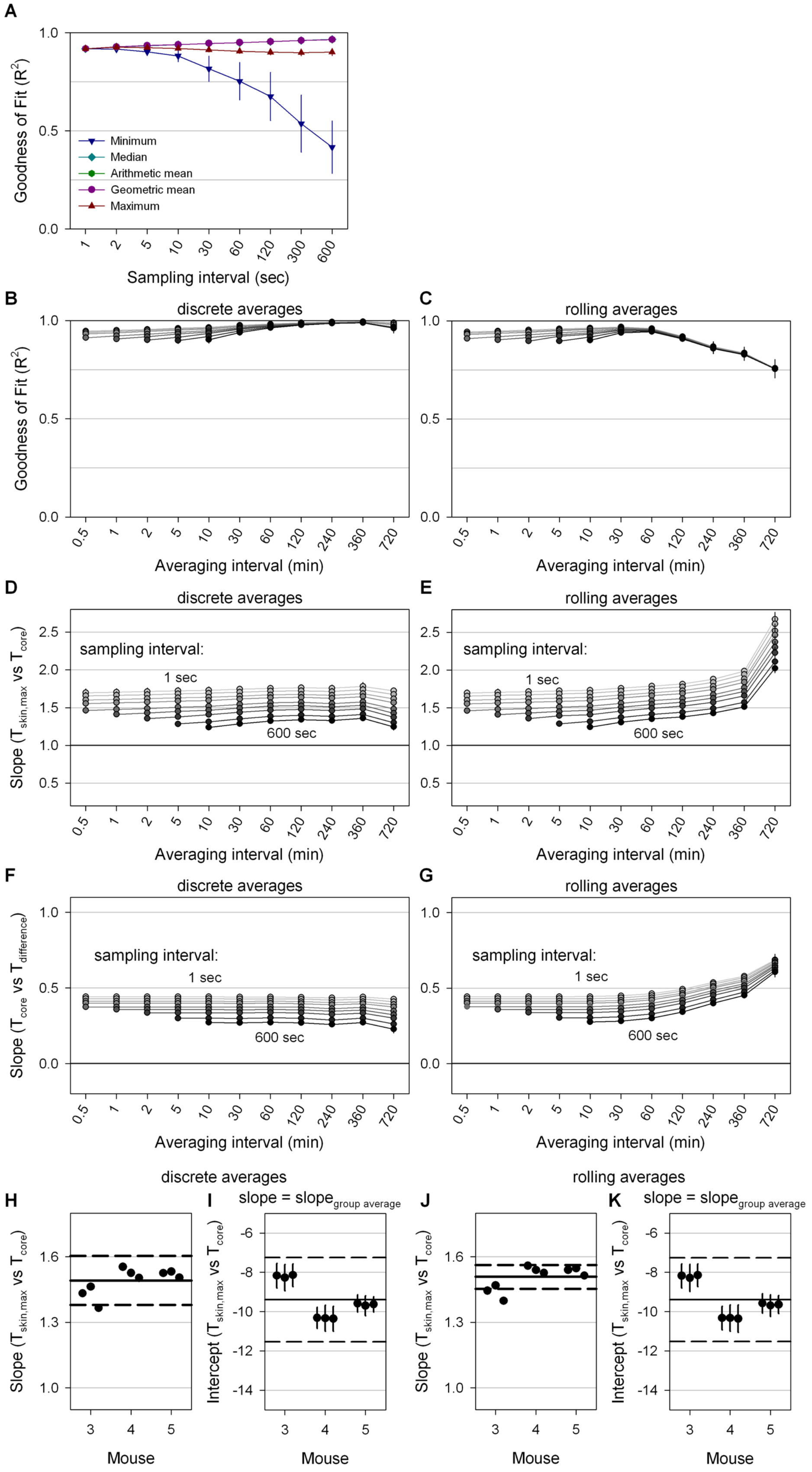

A
Goodness of Fit (R^2)
Minimum
Median
Arithmetic mean
Geometric mean
Maximum
Sampling interval (sec)
B
discrete averages
C
rolling averages
Averaging interval (min)
D
discrete averages
E
rolling averages
Slope ($T_{skin,max}$ vs T_{core})
sampling interval:
1 sec
600 sec
F
discrete averages
G
rolling averages
Slope (T_{core} vs $T_{difference}$)
H
discrete averages
I
slope = $slope_{group\ average}$
Intercept ($T_{skin,max}$ vs T_{core})
J
rolling averages
K
slope = $slope_{group\ average}$
Mouse

Figure S7: Core temperature estimation by continuous skin temperature measurements during daily torpor; parameter optimisation. (A) Goodness of fit associated with different summary statistics calculated over intervals between 1 sec and 10 min. (B) Goodness of fit associated with discrete estimates of T_{core} for each averaging interval based on $T_{skin,max}$ over different sampling intervals (1-600 sec) and averaged over intervals between 30 sec and 12 hours. (C) Goodness of fit associated with estimating each measurement of T_{core} (30 sec time resolution) using a rolling average based on $T_{skin,max}$ over different sampling intervals (1-600 sec) and averaged over intervals between 30 sec and 12 hours. (D, E) The slope describing the relationship between $T_{skin,max}$ and T_{core} depends on the sampling- but not the averaging interval. (F, G) The slope describing the relationship between T_{core} and the difference between $T_{skin,max}$ and T_{core} depends on the sampling interval but not on the averaging interval. (H, J) The slope of the linear relationship between T_{core} and $T_{skin,max}$ in all three mice on the three assessment days. (I, K) The intercept of the linear relationship between T_{core} and $T_{skin,max}$ in all three mice on the three assessment days. This assessment used the group-average as the slope for all mice. Solid lines in (H, K) represent the group mean while dashed lines enclose the 2-standard-deviations area surrounding this average. Calculations were based on discrete averages (B, D, F, H, I) or rolling averages (C, E, G, J, K). Error bars represent between-animal SEM (A-G) or within-animal within-day SD (I, K). Fill and line colour become progressively darker with increasing sampling interval duration (B-G).

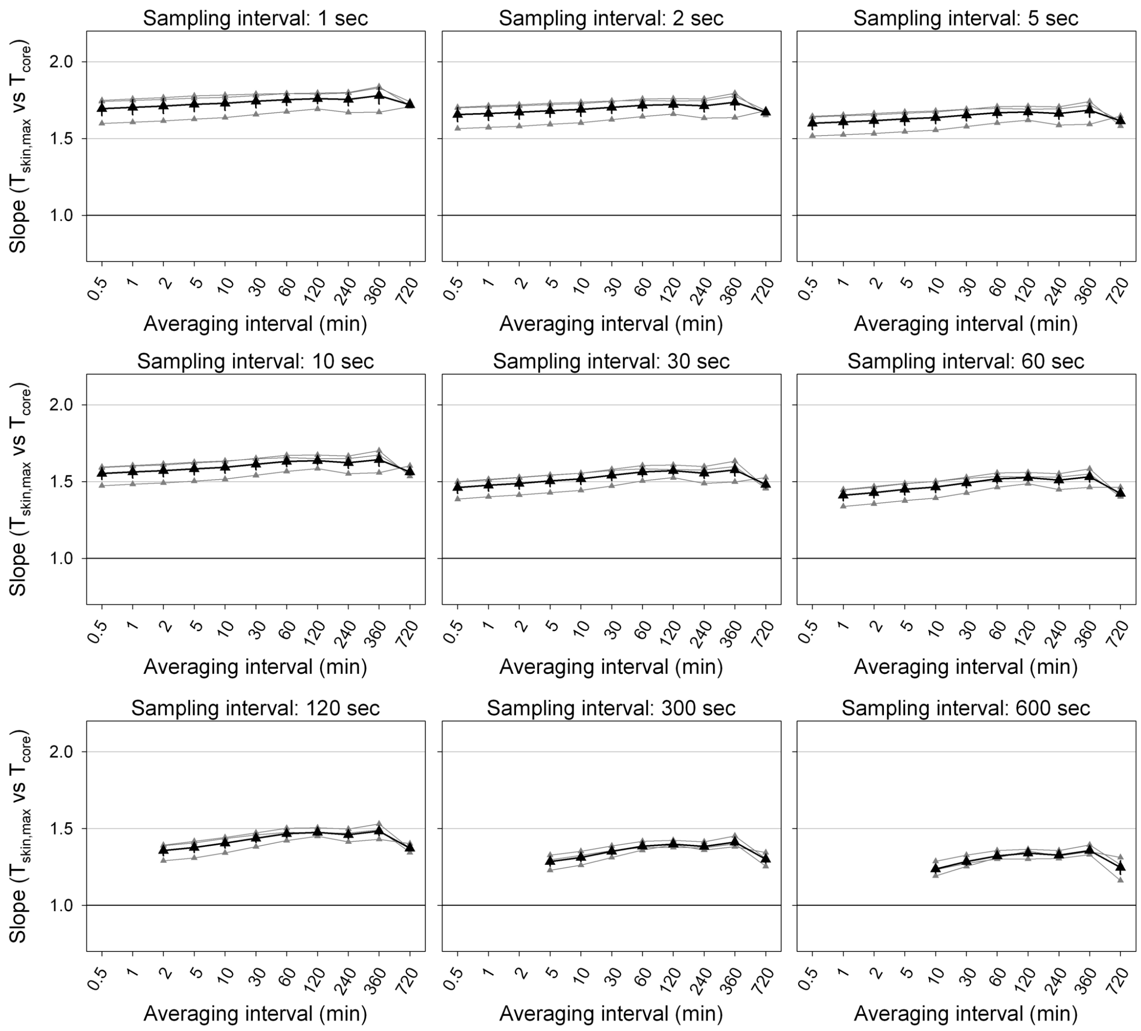

Figure S8: The slope describing the relationship between $T_{skin,max}$ and T_{core} in energetically-challenged mice exhibiting daily torpor. Dark-grey lines represent the observed slope in each of the three mice. Black lines represent group averages and are identical to data presented in S7D. $T_{skin,max}$ per sampling interval was used as the summary statistic and all slopes were based on analysis of the discrete averages.
